# Magnitude and patterns of severe *Plasmodium vivax* monoinfection in Vietnam: a 4-year single-center retrospective study

**DOI:** 10.3389/fmed.2023.1128981

**Published:** 2023-05-30

**Authors:** Minh Cuong Duong, Oanh Kieu Nguyet Pham, Thanh Truc Thai, Rogan Lee, Thanh Phong Nguyen, Van Vinh Chau Nguyen, Hoan Phu Nguyen

**Affiliations:** ^1^School of Population Health, University of New South Wales, Sydney, NSW, Australia; ^2^Hospital for Tropical Diseases, Ho Chi Minh City, Vietnam; ^3^Faculty of Public Health, University of Medicine and Pharmacy at Ho Chi Minh City, Ho Chi Minh City, Vietnam; ^4^Centre for Infectious Diseases and Microbiology, Pathology West-ICPMR and Marie Bashir Institute, University of Sydney, Westmead Hospital, Westmead, NSW, Australia; ^5^Department of Health, Ho Chi Minh City, Vietnam; ^6^Centre for Tropical Medicine and Global Health, Nuffield Department of Medicine, University of Oxford, Oxford, United Kingdom; ^7^Medical School, Vietnam National University of Ho Chi Minh City, Ho Chi Minh City, Vietnam; ^8^Oxford University Clinical Research Unit (OUCRU), Ho Chi Minh City, Vietnam

**Keywords:** *Plasmodium vivax*, monoinfection, severe malaria, delayed hospital admission, hospital length of stay, Vietnam

## Abstract

**Introduction:**

Infection with *Plasmodium vivax* is a recognized cause of severe malaria including deaths. The exact burden and patterns of severe *P. vivax* monoinfections is however still not well quantified, especially in *P. vivax* endemic regions. We examined the magnitude and patterns of severe malaria caused by monoinfections of *P. vivax* and associated predictors among patients admitted to a tertiary care center for malaria in Vietnam.

**Methods:**

A retrospective cohort study was conducted based on the patients’ medical records at the Hospital for Tropical Diseases from January 2015 to December 2018. Extracted information included demographic, epidemiologic, clinical, laboratory and treatment characteristics.

**Results:**

Monoinfections with *P. vivax* were found in 153 (34.5, 95% CI 30.3–39.1%) patients of whom, uncomplicated and severe malaria were documented in 89.5% (137/153, 95% CI 83.7–93.5%) and 10.5% (16/153, 95% CI 6.5–16.3%), respectively. Patterns of severe malaria included jaundice (8 cases), hypoglycemia (3 cases), shock (2 cases), anemia (2 cases), and cerebral malaria (1 case). Among 153 patients, 73 (47.7%) had classic malaria paroxysm, 57 (37.3%) had >7 days of illness at the time of admission, and 40 (26.1%) were referred from other hospitals. A misdiagnosis as having other diseases from malaria cases coming from other hospitals was up to 32.5% (13/40). Being admitted to hospital after day 7th of illness (AOR = 6.33, 95% CI 1.14–35.30, p = 0.035) was a predictor of severe malaria. Severe malaria was statistically associated with longer hospital length of stay (p = 0.035). Early and late treatment failures and recrudescence were not recorded. All patients recovered completely.

**Discussion:**

This study confirms the emergence of severe vivax malaria in Vietnam which is associated with delayed hospital admission and increased hospital length of stay. Clinical manifestations of *P. vivax* infection can be misdiagnosed which results in delayed treatment. To meet the goal of malaria elimination by 2030, it is crucial that the non-tertiary hospitals have the capacity to quickly and correctly diagnose malaria and then provide treatment for malaria including *P. vivax* infections. More robust studies need to be conducted to fully elucidate the magnitude of severe *P. vivax* in Vietnam.

## 1. Background

Despite a recent dramatic reduction in the number of infected cases and deaths, malaria remains a global public health problem (1, 2). It was estimated that there were 247 million cases of malaria and 619,000 malaria-related deaths worldwide in 2021 (3). *Plasmodium falciparum, P. vivax, P. ovale, P. malariae,* and *P. knowlesi* are the five species that cause malaria in humans of which, *P. falciparum* is responsible for the majority of malaria infections and malaria-related deaths (1, 4). However, there has been an increasing focus towards the burden of *P. vivax* infection (5–8). In the endemic regions with co*-*existence of *P. falciparum* and *P. vivax*, there is a decrease in *P. falciparum* infections due to successful malaria control programs. *P. vivax* has now become the predominant species of infection (9). In addition, several reports worldwide indicate that *P. vivax* can cause severe malaria like *P. falciparum* (5–8, 10–12). Thus, *P. vivax* is not only a cause of benign malaria infection, but can also be a cause of severe infections in patients who received delayed treatment (8, 11, 12).

Although *P. vivax* is the most geographically widespread Plasmodium species causing human disease, the common low-density *P. vivax* infection poses a challenge for *the* elimination of this malaria species (13, 14). The current *P. falciparum* control and elimination strategies cannot simply be used for *P. vivax* due to the unique biology of this Plasmodium species, such as the ability to induce dormant liver-stage infections responsible for the hypnozoite reservoir of *P. vivax* infections (15). Therefore, developing a more specific *P. vivax* control and elimination strategy is needed (15). It has been recommended that clinical characterization of severe *P. vivax* infection is crucial in further understanding its pathogenesis, which will subsequently provide better treatment outcomes with *P. vivax* infections (16). The World Health Organization (WHO) describes, in addition to uncomplicated malaria, 11 different, severe manifestations of *P. vivax* infection as seen in *P. falciparum* infections but without reaching similar parasite density thresholds (17). These manifestations include impaired consciousness, prostration, multiple convulsions, acidosis, hypoglycemia, severe malarial anemia, renal impairment, jaundice, pulmonary edema, significant bleeding, and shock (17). However, despite these recognized severe manifestations and a recent recognition that *P. vivax* can cause deaths, there are still insufficient data that quantify the exact burden and patterns of severe *P. vivax* monoinfections, especially in *P. vivax* endemic regions (6).

Vietnam is located in Southeast Asia, in which the most prevalent malaria species are *P. falciparum* and *P. vivax* (18–20). The most recent WHO’s statistics shows that a total of 381 malaria confirmed cases were reported in Vietnam in the first 10 months of 2022 (21). Of these cases, *P. vivax* accounted for 39% (21). However, it should be noted that the magnitude of *P. vivax* changes over time. Indeed, *P. vivax* accounted for 57% (75/131) of a total of 131 malaria confirmed cases in the first half of 2022 (22), demonstrating an important role of this parasite species in the burden of malaria in Vietnam (22). Like other countries, research on malaria in Vietnam has been impacted by the COVID-19 pandemic. Indeed, it has been well documented that in comparable developing countries, *“efforts to control COVID-19 would impact efforts to control other existing health problems that are endemic, such as malaria”* (23). To the best of our knowledge, there are a few papers related to malaria in Vietnam published between 2020 and 2022 (18, 19, 24–27). However, none of these studies specifically documented the evolution of malaria in southern Vietnam. There was also no study focusing on exploring the patterns of severe vivax malaria and associated risk factors which have implications for improving patient care and disease prevention. We aimed to examine the magnitude and patterns of cases with severe malaria caused by monoinfections of *P. vivax* and associated predictors among patients admitted to a tertiary care center for malaria in Vietnam.

## 2. Methods

### 2.1. Study context

The Hospital for Tropical Diseases (HTD) located in Ho Chi Minh City is among the few tertiary teaching hospitals for infectious diseases including malaria in Vietnam. HTD is also a severe malaria referral hospital in southern Vietnam. As per HTD policy, patients’ information including medical history, epidemiology, diagnosis, and treatment must be correctly entered into the medical records. All malaria related laboratory tests including microscopy and rapid tests are performed at the HTD and in accordance with the national laboratory performance standards. All suspected cases are primarily diagnosed using microscopy, followed by screenings of all clinical and laboratory signs of severe malaria. Microscopy is subsequently performed at an interval of six or 12 h, depending on the disease severity until it turns into a negative result for at least two times. Rapid test is performed based on the physicians’ clinical judgement. According to the Vietnam Ministry of Health guidelines for the management of malaria infection, in order to be discharged from the hospital, patients must meet the following four criteria: receiving a full 3-day course of chloroquine of 25 mg (base)/kg or an oral artemisinin-based combination therapy, no fever, no severe malaria symptoms, and blood smear negative for parasites, excluding gametocytes stages, for at least two times (28). Patients receive a 14-day primaquine regimen of 0,25 mg (base)/kg/day when they are confirmed not to have G6PD deficiency and continue this regimen even after discharge. Patients are advised to seek medical attention if fever returns.

### 2.2. Study design

A 4-year retrospective cohort study was conducted based on the patients’ medical records at the HTD from January 2015 to December 2018. The study was approved by the HTD’s Ethics Committee (reference number 65/QD-BVBND) and UNSW Sydney’s Human Research Ethics Committee (reference number HC180340). Medical records of *P. vivax* infected patients were used to extract information on the patients’ demographics, malaria related risk factors, pregnancy status (for female patients), underlying health conditions, history of malaria infection, current course of malaria disease and treatments at previous hospitals and HTD. To ensure the validity of data, two authors (OP and TN) who are qualified infectious disease specialists at the HTD reviewed and extracted data from the medical records. Any discrepancies derived from this process were cross-checked until consensus was obtained.

Demographic information included age, sex, job, residential address, and BMI. In addition, malaria related risk factors comprising of previous blood transfusions (transfusions within 1 month piror to the onset of disease), injecting drug use (IDU), living in or traveling to malaria endemic areas within the 7 days prior to the onset of disease, and previous history of malaria infection. Underlying health conditions included end stage renal disease, cirrhosis, and other chronic diseases. Information on the current course of malaria disease included admission time, number of days of illness at the time of admission, signs and symptoms (fever, anemia, jaundice, shock, consciousness, splenomegaly, and hepatomegaly), laboratory tests (malaria microscopy and rapid test (SD Bioline Malaria Ag P.f/P.v, Standard Diagnostics, Inc., South Korea), aminotransaminases (AST and ALT), arterial blood gas, hemoglobinuria, and serum levels of electrolytes, creatinine, glucose, bilirubin, and lactate), abdominal sonography. Treatments included anti-malarial and other supportive treatments, response to treatment (number of inpatient days, fever clearance time, parasite clearance time, early (ETF) and late treatment failure (LTF)), recrudescence, and outcomes (recovery and death). Diagnosis and treatment at previous hospitals were also documented.

### 2.3. Definitions of early and late treatment failures

ETF and LTF were defined in line with the WHO recommendations (29). ETF included (i) danger signs or severe malaria on day 1, 2 or 3, in the presence of parasitemia; or (ii) parasitemia on day 2 higher than on day 0, irrespective of axillary temperature; (iii) parasitemia on day 3 ≥ 25% of count on day 0; and (iv) parasitemia with axillary temperature ≥37.5°C on day 3. The presence of ETF among study participants was examined based on the clinical symptoms and laboratory tests. Regarding LTF, it was defined as (i) danger signs or severe malaria in the presence of parasitemia on any day between day 4 and day 28 (or day 42) in patients who did not previously meet any of the criteria of ETF; or (ii) presence of parasitemia on any day between day 4 and day 28 (or day 42) with axillary temperature ≥37.5°C in patients who did not previously meet any of the criteria of ETF. Our patients with malaria are monitored for up to 28 days post treatment, and this information is documented in the medical record as required by the HTD policy. Therefore, patients with LTF in the presenting study were those who met the WHO definition of LTF within 28 days before admission.

### 2.4. Statistical analysis

Data were managed and analyzed using SPSS version 26 (IBM Corp, Armonk, NY). For comparison purposes, 95% confidence intervals (CI) of the point incidence of uncomplicated and severe *P. vivax* monoinfections were calculated based on the total number of patients with *P. vivax* monoinfection and the point estimate of the incidence of uncomplicated and severe *P. vivax* monoinfections. Descriptive statistics were performed to summarize characteristics of study participants. Categorical variables were presented as an absolute count and percentage, while continuous variables were presented as mean ± 1 standard deviation (SD). Inferential statistics including Fisher’s exact test and Student’s t-test were used to compare categorical and continuous data, respectively, that facilitate the identification of predictors of severe malaria. To test predictors of severe malaria, a multivariable logistic regression model was developed. Based on the purposeful selection process, covariates for the regression model were identified and included variables that have a *p* value <0.25 in the univariable analysis as well as those that are clinically important based on the authors’ judgement (30, 31). In contrast, variables that had a large number of missing values or with a low frequency were excluded from the regression model (30, 31). Variables included in the regression model were age, gender, BMI, history of malaria infection, living in or traveling to malaria endemic areas, previous blood transfusion and underlying health conditions, being diagnosed with malaria infection at previous hospitals, and hospital admission after day 7th of illness. Alpha was set at 5% level.

## 3. Results

### 3.1. Baseline characteristics of 153 study participants

During the study period, the HTD received a total of 443 malaria infected patients including 153 (34.5, 95%CI 30.3–39.1%) with *P. vivax* monoinfection. Of these 153 patients, males and females accounted for 80.4% (123/153) and 19.6% (30/153), respectively (Table 1). The mean age and BMI of these *P. vivax* infected patients was 33.4 ± 11.7 years old and 21.5 ± 3, respectively. More than two-thirds (73.2%, 112/153) of them resided in or traveled to malaria-endemic areas 7 days prior to the onset of malaria. These participants included 52 people worked in forest-related fields. Around one-third (32.7%, 50/153) of them reported having a previous history of a malaria infection. All these 50 patients previously acquired malaria infections more than 12 months prior to the current illness. Three patients reported having at least one previous blood transfusion. Among 30 female patients, three were pregnant. Chronic kidney disease, cirrhosis and injecting drug use were not documented. Among the 153 patients with *P. vivax* monoinfection, 57 (37.3%) of them had more than 7 days of illness at the time of admission. Forty patients (26.1%) were transferred from previous hospitals. Of these 40 patients, 13 had a wrong diagnosis of having other diseases. The remaining 27 patients were correctly diagnosed as having vivax malaria infection. Four (10%) of these transferred patients received antimalarial treatment at previous hospitals. Among four patients receiving antimalarials at previous hospitals, one received oral DHA/PPQ for 1 day, while another patient used chloroquine and DHA/PPQ for 1 day. The remaining two patients used chloroquine and primaquine for one and 2 days, respectively. Parasitemia still persisted in all of them at the time of admission.

**Table 1:** Baseline characteristics of 153 patients with single *Plasmodium vivax* infection.

Characteristics	Summary statistics*
Age	33.4 ± 11.7
Male	123 (80.4)
Forest-related jobs**	52 (40.5)
Living in or travelling to malaria-endemic areas 7 days prior to the onset of disease	112 (73.2)
History of malaria infection	50 (32.7)
BMI	21.5 (3.0)
Injecting drug use	0
Pregnancy	3 (2.0)
*Previous blood transfusion and underlying health conditions*	
Previous blood transfusion	3 (2.0)
End stage renal disease	0
Cirrhosis	0
Other***	3 (2.0)
*Diagnosis at previous hospitals (n = 40)*	
Vivax malaria	27 (67.5)
Dengue infection	6 (15.0)
Septicemia	1 (2.5)
Others****	6 (15.0)
*Receiving treatment at previous hospital (n = 27)*	
Antimalarial treatment	4 (14.8)
Supportive treatment	11 (40.7)
No treatment	12 (44.4)
Hospital admission after day 7th of illness	57 (37.3)

* *N* (%) for categorical variables and Mean ± SD for continuous variables.

** Mountain farmers, forest workers, forest ranger, healthcare worker working in the forest.

*** Idiopathic thrombocytopenic purpura, sick sinus syndrome with an implanted pacemaker, type 2 diabetes mellitus.

**** Typhoid fever and viral infection.

### 3.2. Clinical and laboratory characteristics, malaria infection classification, treatments, and outcomes

All patients had fever on admission (Table 2). The most common clinical manifestations were: classic malaria paroxysm (47.7%, 73/153), followed by hepatomegaly (4.6%, 7/153), jaundice (3.3%, 5/153), and splenomegaly (2.6%, 4/153). Among 84 patients undertaking a rapid malaria test, 94% (79/84) tested positive. Among all 153 patients, AST >40 U/L was recorded in 11.1% (17/153), ALT >40 U/L in 16.3% (25/153), total bilirubin >50 umol/l in 5.2% (8/153), G6PD deficiency in 3.3% (5/153), and hemoglobinuria was not documented. Among 70 patients who underwent abdominal sonography, 10% (7/70) showed splenomegaly, while both splenomegaly and hepatomegaly were recorded in 38.6% (27/70). Uncomplicated malaria was documented in 89.5% (137/153, 95% CI 83.7–93.5%) of patients, while 10.5% (16/153, 95% CI 6.5–16.3%) had severe malaria. All these 16 patients with severe malaria had a single clinical manifestation such as shock (two cases), cerebral malaria (one case), jaundice (eight cases), anemia (two cases), and hypoglycemia (three cases).

**Table 2:** Clinical and laboratory characteristics, and malaria infection classification of 153 study participants.

Characteristics	Summary statistics^*^
**Clinical characteristics**	
*Signs and symptoms*	
Fever on admission	153 (100)
Classic malaria paroxysm	73 (47.7)
Hepatomegaly	7 (4.6)
Jaundice	5 (3.3)
Splenomegaly	4 (2.6)
Shock	2 (1.3)
Impaired consciousness (GCS <13)	1 (0.7)
**Laboratory characteristics**	
Positive rapid test (*n* = 84)	79 (94.0)
AST > 40 U/L	17 (11.1)
ALT > 40 U/L	25 (16.3)
Hyperkalemia (>4.5 mmoL/L)	1 (0.7)
Serum creatinine (umol/L)	79.4 ± 18.9
Plasma glucose (mmol/l)	6.7 ± 2.5
Hemoglobinuria	0
Total bilirubin >50 umol/l	8 (5.2)
Lactate >4 mmol/L (*n* = 16)	1 (6.3)
pH < 7.35 and HCO_3_ < 15 (*n* = 16)	0
G6PD deficiency	5 (3.3)
**Imaging test (Abdominal ultrasound) (*n* = 70)**	
Splenomegaly	7 (10.0)
Splenomegaly and hepatomegaly	27 (38.6)
**Malaria classification**	
Uncomplicated malaria	137 (89.5)
Severe malaria	16 (10.5)
Shock	2 (1.3)
Cerebral malaria	1 (0.7)
Jaundice	8 (5.2)
Hypoglycemia	3 (2.0)
Anemia	2 (1.3)

* *N* (%) for categorical variables and Mean ± SD for continuous variables.

Regarding antimalarial treatment, among 137 patients with uncomplicated malaria, 112 were treated with chloroquine, while 25 were given DHA/PPQ (Table 3). Among the 16 patients with severe malaria, 11 received chloroquine. The remaining five severe cases were given parenteral artesunate, of whom four and one subsequently switched to chloroquine and DHA/PPQ, respectively. In addition to anti-malarial treatment, 1.3% (2/153) of patients received red blood cell transfusion. Hospital acquired infection was not recorded in any patient. The proportion of patients with fever after 72 h of antimalarial treatment was 4.6% (7/153), and that of patients with parasitemia after 72 h of antimalarial treatment was 2% (3/153). There was no association between persistent fever and parasitemia after 72 h of antimalarial treatment and severe vivax malaria (*p* > 0.05; Appendix 1). There was no patient with parasitemia after 7 days of antimalarial treatment. ETF and LTF as well as recrudescence were also not recorded. The mean number of inpatient days was 4.5 ± 1.8. The mean number of inpatient days of those with severe malaria was significantly higher than that of those with uncomplicated malaria (6.19 ± 3.12 vs. 4.36 ± 1.54, *p* = 0.035, data not shown). All patients completely recovered.

**Table 3:** Treatments and outcomes of 153 study participants.

Characteristics	Summary statistics*
**Treatments**	
*Anti-malarial treatment*	
Uncomplicated malaria (*n* = 137)	
Chloroquine	112 (81.8)
Dihydroartemisinin-piperaquine	25 (18.2)
Severe malaria (*n* = 16)	
Chloroquine	11 (68.8)
Artesunate	5 (31.2)
*Supportive treatment*	
Mechanical ventilation	0
Red blood cell transfusion	2 (1.3)
Hemodialysis	0
**Outcomes**	
Number of inpatient days	4.5 ± 1.8
Recovery	153 (100)
Patients with fever after 3 days of anti-malarial treatment	7 (4.6)
Patients with parasitemia after 72 h of anti-malarial treatment	3 (2.0)
Patients with parasitemia after 7 days of anti-malarial treatment	0
Early treatment failure	0
Late treatment failure	0
Recrudescence	0

* *N* (%) for categorical variables and Mean ± SD for continuous variables.

### 3.3. Association between baseline characteristics and severe *Plasmodium vivax* monoinfection

There was an association between severe malaria and age (39.3 ± 11.9 years vs. 32.7 ± 11.5 years, *p* = 0.033), being diagnosed with malaria infection at previous hospitals (OR = 3.31, 95% CI 1.09–13.09, *p* = 0.039) and being admitted to hospital after day 7th of illness (OR = 6.13, 95% CI 1.87–20.09, *p* = 0.001; Table 4). There was no statistically significant association between severe malaria and sex, BMI, previous blood transfusion and underlying health conditions, previous infection with malaria, and living in or traveling to malaria endemic areas (*p* > 0.05).

**Table 4:** Association between demographic and clinical characteristics and severe *Plasmodium vivax* infection among 153 study participants.

Characteristics	Severe malaria*		*p* value**	OR (95% CI)
	(+) (*n* = 16)	(−) (*n* = 137)		
Age	39.3 ± 11.9	32.7 ± 11.5	0.033	
BMI	21.1 ± 3.0	21.5 ± 3.0	0.640	
Living in or travelling to malaria endemic areas	10 (62.5)	102 (74.5)	0.371	0.57 (0.19–1.69)
Being diagnosed with malaria infection at previous hospitals	6 (37.5)	21 (15.3)	0.039	3.31 (1.09–13.09)
Male	11 (68.8)	112 (81.8)	0.314	0.49 (0.16–1.54)
History of malaria infection	2 (12.5)	48 (35)	0.069	0.26 (0.06–1.21)
Pregnancy	1 (6.3)	2 (1.5)	0.284	4.50 (0.38–52.62)
Previous blood transfusion and underlying health conditions	1 (6.3)	5 (3.6)	0.491	1.76 (0.19–16.08)
Hospital admission after day 7th of illness	12 (75)	45 (32.8)	0.001	6.13 (1.87–20.09)

* *N* (%) for categorical variables and Mean ± SD for continuous variables.

** Fisher’s exact test for categorical variables and Student’s *t-*test for continuous variables.

### 3.4. Model for predicting severe *Plasmodium vivax* monoinfection

No other predictors for severe *P. vivax* infection were identified other than being admitted to hospital after day 7th of illness (AOR = 6.33, 95%CI 1.14–35.30, *p* = 0.035; Appendix 2).

## 4. Discussion

Surveillance data indicated that *P. falciparum* was the predominant species in Vietnam and accounted for 70% of malaria infections from 2006 to 2010 (32). However, *P. vivax* has become an important concern in recent years due to the successful control efforts against *P*. *falciparum* (22, 32). According to the 2022 World Malaria Report, despite some fluctuations, the ratio of falciparum malaria to vivax malaria sharply dropped from 2.9:1 in 2010 to 0.6:1 in 2021 (3). Our study shows that 34.5% (95% CI 30.3–39.1%) of all 443 confirmed malaria infected patients admitted to hospital during the study period were *P. vivax* monoinfections. In light of this, despite a decrease in the absolute number of malaria cases in Vietnam in recent years, the proportion of cases due to *P. vivax* remains high.

We also noted that most patients lived in or traveled to malaria-endemic areas 7 days prior to the onset of malaria or worked in forest-related fields. Indeed, it has been well documented that the rural forest areas in the central highlands of Vietnam and along the international borders of Laos and Cambodia with Vietnam are hyper-endemic for malaria (33, 34). Those workers who enter forests are at higher risk of acquiring malaria infections (32).

The presenting study found that 10.5% (95%CI 6.5–16.3%) had severe *P. vivax* malaria. In Vietnam, information about severe *P. vivax* is scarce. Our rate of severe cases was higher than that reported in a systematic review of clinical studies on severe vivax malaria published between 1900 and 2014, in which the pooled prevalence of severe vivax malaria ranged from 0.5 to 4.7% (35). Our finding highlights the increasing impact of vivax malaria and the potential of developing sever disease as falciparum malaria infections decline. Our rate of severe cases is comparable with that of a more recent study conducted in Korea but lower than that of an Ethiopian study (36, 37). Although the reason for this remains unclear, several studies conducted in India showed the magnitude of severe *P. vivax* ranging from 8.8 to 78.9% demonstrating a diverse burden of this disease across a given country (6, 38–40). Since our study was a single-center study, we believe that broader studies are needed to fully understand the burden of severe *P. vivax* in Vietnam.

Recent studies have identified the emergence of severe *P. vivax* in Asia, and an increasing burden of morbidity and mortality associated with this malaria species has also been reported (12, 41–43). The patterns of severe *P. vivax* malaria among our study participants included jaundice (eight cases), hypoglycemia (three cases), shock (two cases), anemia (two cases), and cerebral malaria (one cases). These findings concurred with other studies (6, 8, 40, 44), although we did not record any acute lung injury which is commonly reported to be associated with severe *P. vivax* malaria (15). Jaundice is considered to be the most common manifestation of severe *P. vivax* infection and common cause of hospitalization in some regions (8). However, it has been indicated that hyperbilirubinemia (total bilirubin >3.0 mg/dL) is a weak marker of severity (8). In contrast, shock has been frequently reported in patients who die from severe *P. vivax* suggesting that it is a good marker of severity (8). Cerebral malaria is classically the most lethal complication of infection with *P. falciparum* malaria and has increasingly reported to be associated with *P. vivax* infections (8, 40, 44). Although this manifestation is infrequent in our participants, as indicated in a systematic review (8), the detection of neurological symptoms in our study emphasized the importance of ruling out other infections such as bacterial or viral meningoencephalitis as well as malarial complications such as hypoglycemia and metabolic acidosis that can cause misdiagnosis. Regarding treatment outcomes, despite a complete recovery of all study participants, those with severe vivax malaria had a longer hospital length of stay compared to those with uncomplicated malaria. This highlights the key role of early and accurate diagnosis of malaria and severe malaria in effective disease management.

Antimalarial drug resistance has been a major concern in managing clinical malaria. Chloroquine is the first-line treatment for *P. vivax* monoinfection in Vietnam and other endemic countries due to its effectiveness and low cost (45). However, chloroquine-resistant *P. vivax* has been reported in several endemic regions in Southeast Asia (15, 46). In Vietnam, surveillance of *P. vivax* resistance to chloroquine remains challenging due to the lack of resources and difficulties in differentiating between real treatment failure and reinfection or liver relapse (24). Treatment with either chloroquine, DHA/PPQ or artesunate or a combination of these drugs in our patients with severe malaria resulted in total clearance of the parasite. Unfortunately, ETF and LTF were not documented in this study. To the best of our knowledge, artemisinin-resistant *P. vivax* parasites have not been documented in Vietnam. However, limited data in Vietnam show that *P. vivax* resistance to chloroquine was first recorded in Binh Thuan province (southeastern coast region) in the early 2000s (47), and subsequently in the remote forested area of Quang Nam province (central Vietnam) in 2015 (48), and in Ninh Thuan province (south-central Vietnam) in 2019 (49). In 2021, a WHO-recommended therapeutic efficacy study of chloroquine against uncomplicated *P. vivax* malaria conducted on 67 patients in Gia Lai province (central Vietnam) confirmed that chloroquine remained largely efficacious to treat this infection with an adequate clinical and parasitological response rate of 100% on day 28 (24). Parasitemia after 3 days of antimalarial treatment has been well documented to be associated with chloroquine-resistant *P. vivax* infection (45). Similarly, fever clearance time is a crucial marker for evaluating the effectiveness of chloroquine in treating *P. vivax* infection (50). Hence, the low rates of persistent fever and parasitemia after 3 days of antimalarial treatment in our study also demonstrate that chloroquine remains effective in Vietnam. In addition, the WHO’s study also found that recurrences occurred late (>day 28) and in association with low blood chloroquine concentrations, and on day 42, the recurrence rate was 24.6% (24). A large systematic review and meta-analysis of the effect of chloroquine dose on *P. vivax* recurrence on 2,990 patients from 17 countries treated with chloroquine alone including 1,041 (34·8%) receiving a dose below the recommended dose of 25 mg/kg similarly found that the risk of recurrence was 32.4% by day 42 (51). These studies indicate that chloroquine-resistant *P. vivax* malaria is spreading in Vietnam. Although treatment failures were not recorded in our study, we believe that a national surveillance program is crucial to fully capture the impact of *P. vivax* resistance to chloroquine in Vietnam. In addition, given that elimination of all malaria infections in Vietnam will solve the problems of patients getting severe malaria (52), the spread of antimalarial drug resistance including *P. vivax* chloroquine resistance represents a potential hurdle for achieving this goal.

Risk factors for severe *P. vivax* infection have not been well established, except the presence of co-morbidities, coinfection with *P. falciparum,* and pregnancy (15, 53). Although these risk factors were not documented in our study, we found that being admitted to hospital after day 7th of illness was a predictor of severe *P. vivax* infection in our study population. Delayed admission to hospital may partially be due to the primarily asymptomatic *P. vivax* infections (54, 61). In addition, it has been documented that self-medication practices are common among Vietnamese residents, especially those living in highland provinces including endemic areas of malaria infection (55). This practice may cause delays in correct diagnosis and treatment among patients with symptomatic infections. Consequently, community education programs about malaria infections should be strengthened to focus on early symptoms and correct management of malaria to prevent delayed treatment and development of severe *P. vivax* infections. In addition, we also noticed that among 40 patients diagnosed at previous hospitals, one third of them were incorrectly diagnosed as having other diseases rather than malaria at previous hospitals. It has been well documented that insufficient malaria diagnostic capacity of healthcare facilities is the most important risk factor for misdiagnosis (56). Given that the classic malaria paroxysm is the hallmark symptom of malaria (57), the misdiagnoses could be partially due to the atypical manifestations of vivax malaria demonstrated by less than half of our participants having a classic malaria paroxysm. In addition, signs and symptoms of malaria are similar to those of other febrile illnesses that are endemic in Vietnam such as dengue fever, typhoid fever, and respiratory tract infection (57). It has been documented that in rural areas where parasitological tests for malaria are not available, the complexity of malaria diagnosis may lead to misdiagnosis (57). Indeed, despite eight severe vivax malaria patients showing jaundice, only five of them were identified by clinical examination in our study. In addition, we found that 73.2% of our study participants lived in or traveled to malaria-endemic areas within the 7 days prior to the onset of disease. Inadequate history-taking including questions on the patients’ travel history could make this disease under-diagnosed, especially in areas where incidence is rare (58). Vietnam is recognized as a malaria endemic country, but aims to eliminate this infection by 2030. Strengthening the capacity to diagnose and treat malaria in non-tertiary hospitals will be pivotal in securing this aim (52).

The study has some clear limitations. Firstly, to the best of our knowledge, this is the first study conducted to examine the burden and patterns of severe *P. vivax* monoinfection in Vietnam. However, the study participants were from a single but the largest tertiary teaching hospital in southern Vietnam providing treatment to malaria patients in this area including the Greater Mekong Subregion. Given a single-center study design, our data were limited to those receiving treatment at the HTD. Hence, the study findings might not be representative of the entire Vietnamese population. A single-center study design may also be attributable to the low number of cases in our study. However, our recorded number of cases may proportionally reflect a decrease in the total number of malaria cases nationally. Indeed, it was documented that during the study period between 2015 and 2018, the total number of malaria cases sharply decreased from 9,331 to 4,813 in Vietnam (59). Secondly, the research data collected before the COVID-19 pandemic may not reflect the current decreasing burden of malaria infection including vivax malaria in Vietnam (21). Thirdly, our examination of treatment failures was limited to the first 28 days post-treatment due to the availability of patients’ data in our retrospective cohort study. We were unable to examine the potential recurrences that appeared on day 42 in our patients. Fourthly, previous treatment with anti-malarial drugs and co-infections of *P. vivax* with *P*. *falciparum* may influence the examination of disease severity. Although co-infections were excluded from the analysis, some patients may probably be self-treated with anti-malarial drugs prior to hospital admission but failed to report this to previous hospitals or the HTD. In addition, although the HTD is the leading tertiary hospital specialising in malaria, all malaria infections in our study were diagnosed based on microscopy with an additional rapid test based on the physicians’ clinical judgement, all of which may cause species misdiagnoses. Fifth, given the nature of a retrospective cohort study, we may have missed information on the history of malaria infection among patients who could not fully recall their medical history and lost their paper-based medical records. Sixth, recurrence of vivax malaria includes recrudescence, reinfection, and relapse (60), of which, the last two conditions were not fully differentiated in our study. Indeed, our patients’ recrudescence was detectable due to the strict implementation of the criteria for vivax malaria recovery (28). Reinfection and relapse were also differentiated in patients who no longer lived in an endemic area of malaria post treatment. However, given the 28-day follow-up period post treatment, we were unable to fully detect these two conditions in our patients after this period and in those who continued to live in an endemic area of malaria or subsequently developing subclinical infection and thus, did not seek medical treatment.

In conclusion, this study has provided insight into the burden and patterns of severe *P. vivax* malaria in Vietnam. Although uncomplicated vivax malaria remains common, severe presentations are emerging and associated with being admitted to hospital after day 7th of illness and longer hospital length of stay. The manifestations of *P. vivax* malaria can be misdiagnosed. To meet the goal of malaria elimination by 2030, it is crucial to strengthen the ability to correctly diagnose and treat malaria in non-tertiary hospitals. Community education programs should also focus on early symptoms and correct management of malaria. More robust studies need to be conducted to fully elucidate the magnitude of severe *P. vivax* and *P. vivax* resistance to chloroquine in Vietnam.

## Data availability statement

The raw data supporting the conclusions of this article will be made available by the authors, without undue reservation.

## Ethics statement

The studies involving human participants were reviewed and approved by the Ethics Committee of Hospital for Tropical Diseases in Vietnam (reference number 65/QD-BVBND) and UNSW Sydney’s Human Research Ethics Committee in Australia (reference number HC180340). The patients/participants provided their written informed consent to participate in this study.

## Author contributions

MCD, OKNP, and HPN: conceptualization, formal analysis, and writing original draft. OKNP, MCD, TPN, VVCN, and HPN: data acquisition. MCD, OKNP, TPN, TTT, RL, VVCN, and HPN: reviewing and editing. All authors contributed to the article and approved the submitted version.

## Funding

MCD is funded by the Australian Government through the Australian Alumni Grants Fund. HPN is funded by OURCU Vietnam.

## Conflict of interest

The authors declare that the research was conducted in the absence of any commercial or financial relationships that could be construed as a potential conflict of interest.

## Publisher’s note

All claims expressed in this article are solely those of the authors and do not necessarily represent those of their affiliated organizations, or those of the publisher, the editors and the reviewers. Any product that may be evaluated in this article, or claim that may be made by its manufacturer, is not guaranteed or endorsed by the publisher.
