# Supplementary material for: Magnitude and patterns of severe Plasmodium vivax monoinfection in Vietnam: a 4-year single-center retrospective study
Source: Front Med (Lausanne). 2023 May 30;10:1128981. doi: 10.3389/fmed.2023.1128981 (PMC10265633; doi:10.3389/fmed.2023.1128981)
Supplement: Supplementary file 1 [file Table_1.docx]

Supplementary Material

# Magnitude and patterns of severe *Plasmodium vivax* monoinfection in Vietnam: a 4-year single‐center retrospective study

Minh Cuong Duong*, Oanh Kieu Nguyet Pham*, Thanh Truc Thai, Rogan Lee, Thanh Phong Nguyen, Van Vinh Chau Nguyen, Hoan Phu Nguyen

*These authors contributed equally to the work

Corresponding author: Hoan Phu Nguyen

[phunh@oucru.org](mailto:phunh@oucru.org)

**Appendix 1.** **Association between severe *Plasmodium vivax* monoinfection and fever and parasitemia after 3 days of anti-malarial treatment among 153 study participants**

| **Characteristics** | **Severe malaria**  **N (%)** | | **P value** |
| --- | --- | --- | --- |
|  | **(+)**  **(n = 16)** | **(-)**  **(n = 137)** |  |
| Patients with fever after 3 days of anti-malarial treatment | 1 (6.7) | 6 (5) | 0.57 |
| Patients with parasitemia after 3 days of anti-malarial treatment | 0 (0) | 3 (2.2) | 1 |

*Fisher's exact test
