# Supplementary material for: Magnitude and patterns of severe Plasmodium vivax monoinfection in Vietnam: a 4-year single-center retrospective study
Source: Front Med (Lausanne). 2023 May 30;10:1128981. doi: 10.3389/fmed.2023.1128981 (PMC10265633; doi:10.3389/fmed.2023.1128981)
Supplement: Supplementary file 2 [file Table_2.docx]

Supplementary Material

# Magnitude and patterns of severe *Plasmodium vivax* monoinfection in Vietnam: a 4-year single‐center retrospective study

Minh Cuong Duong*, Oanh Kieu Nguyet Pham*, Thanh Truc Thai, Rogan Lee, Thanh Phong Nguyen, Van Vinh Chau Nguyen, Hoan Phu Nguyen

*These authors contributed equally to the work

Corresponding author: Hoan Phu Nguyen

[phunh@oucru.org](mailto:phunh@oucru.org)

**Appendix 2.** **Multivariable logistic regression analysis for predictors of severe *Plasmodium vivax* monoinfection among 153 study participants**

| **Predictors** | **P value** | **Adjusted OR (95%CI)** |
| --- | --- | --- |
| Age | 0.071 | 1.05 (0.99 – 1.12) |
| BMI | 0.675 | 0.95 (0.74 – 1.22) |
| Being diagnosed with malaria infection at previous hospitals | 0.769 | 1.26 (0.27 – 5.75) |
| Male | 0.575 | 0.65 (0.15 – 2.89) |
| History of malaria infection | 0.626 | 1.67 (0.21 – 13.21) |
| Previous blood transfusion and underlying health conditions | 0.231 | 4.96 (0.36 – 67.90) |
| Hospital admission after day 7^th^ of illness | 0.035 | 6.33 (1.14 – 35.30) |
